# Age-Related Use and Perceptions of eHealth in Men With Prostate Cancer: A Web-Based Survey

**DOI:** 10.2196/cancer.4178

**Published:** 2015-06-25

**Authors:** Camella J Rising, Nadine Bol, Gary L Kreps

**Affiliations:** ^1^ Center for Health and Risk Communication Department of Communication George Mason University Fairfax, VA United States; ^2^ Amsterdam School of Communication Research (ASCoR) Department of Communication Science University of Amsterdam Amsterdam Netherlands

**Keywords:** consumer health information, prostate cancer, age groups, information-seeking behavior, social support, psychosocial aspects

## Abstract

**Background:**

Men with prostate cancer require ample information and support along the continuum of care, and eHealth is one way to meet such critical information and support needs. Currently, evidence about how age influences use and perceptions about prostate cancer eHealth information and support is lacking.

**Objective:**

The aim of this paper is to explore use and perceptions about eHealth among men living with prostate cancer. Specifically, we aimed to analyze men with prostate cancer by age-specific cohorts to identify potential age-related differences in use and perceptions about prostate cancer eHealth information.

**Methods:**

We used survey methodology to examine how men under 65 years old with prostate cancer differ from those aged 65 years old or older in use and perceptions about prostate cancer eHealth information and support (n=289).

**Results:**

We found that men in the younger cohort used the Internet more often to be informed about treatment options (*P*=.04) and to learn more about staging/grading (*P*=.01) than men in the older cohort. Results also showed comparatively greater use of online prostate cancer information for emotional support and encouragement by the younger as compared to the older cohort (*P*=.001). Furthermore, the older cohort reported more negative psychosocial effects of eHealth (eg, more anxious, depressed) than younger men (*P*=.002). We also found that as a result of more frequent Internet use, younger men experienced more positive psychosocial effects (eg, more informed, in control) from accessing information about prostate cancer through eHealth channels (b=-0.10, 95% CI -0.28 to 0).

**Conclusions:**

Men with prostate cancer have different information and support needs; our findings suggest that these needs might vary by age. Future research is needed to unravel age-related factors underlying these differences to be better able to tailor prostate cancer eHealth information to men’s information and support needs.

## Introduction

### Overview

Men with prostate cancer often turn to the Internet to fulfill their information and support needs [1,2]. Research has shown that the Internet helps some individuals with prostate cancer gain and share knowledge and experiences to cope with their illness [3,4]. For these reasons, the Internet has become an important eHealth communication channel for men with prostate cancer [1,2]. eHealth is defined as “health services and information delivered or enhanced through the Internet and related technologies” [5].

Although diagnosed more often in older adulthood, or at the median age of 66 years old [6], diagnosis of prostate cancer among younger men has more than doubled over the past two decades [7,8]. Age at the time of diagnosis of prostate cancer is a meaningful factor to consider given the fact that younger men typically live with the consequences of the disease and treatment for a longer amount of time [9]. On the other hand, older men may experience prostate cancer complicated by age-related comorbidities, such as vascular diseases, other cancers, and infections [10]. Ensuring that appropriate and useful prostate cancer eHealth information is available for audiences of diverse ages and life stages is important, given these considerations.

To deepen the understanding of the reasons for using eHealth information and the perceived psychosocial effects of its use, this paper aims to explore use and perceptions about eHealth among younger and older men living with prostate cancer. We refer to younger and older individuals as men under 65 years old and men 65 years and older, respectively. These two age groups have been found worthy of separate analysis in several studies on adulthood development [11] and disease in adulthood [12]. Moreover, dividing individuals into these two cohorts is justified by the median age at diagnosis of prostate cancer, which is 66 years old [6].

### Use and Experience of Prostate Cancer eHealth

In 2013, nearly 80% of adults aged 45 to 64 years had Internet access compared to a little less than 60% of adults aged 65 and over [13]. Although the gap in use between younger and older adults is narrowing, older adults also use eHealth for cancer information less frequently than their younger counterparts [14,15].

When evaluating prostate cancer eHealth and age, it is important to consider that using the Internet does not necessarily mean that individuals find what they seek online or that they perceive the information in the same way. This is often referred to as eHealth literacy, the ability to seek, find, understand, and act on health information from electronic sources to solve a health problem [16], and eHealth literacy is considered lower among older adults [17]. Moreover, older adults often suffer from a decline in basic abilities, such as cognitive (eg, decreased working memory) and sensory (eg, decreased visual acuity) impairments [18], which makes their user experience online different from adults under 65 years of age.

Considering these age-related differences with respect to Internet use and experience, we expect that when compared to younger men with prostate cancer, older men in our study will use the Internet less frequently in general, as well as less frequently specifically for prostate cancer information and/or support (Hypothesis 1a [H1a]). We also expect that older men will have a more negative experience using the Internet in general and in relationship to prostate cancer information and/or support when compared to their younger counterparts (Hypothesis 1b [H1b]).

### Reasons for Using Prostate Cancer eHealth

Prostate cancer eHealth is as varied as the challenges men with prostate cancer face. Examples include information about cancer staging and grading (Gleason score), available treatments, treatment decision-making tools (nomograms) [19], and more complex eHealth tools that address a variety of information and support needs [20,21]. Such tools may appeal to men’s desire for autonomy and security in their treatment decisions [1].

Other manifestations of prostate cancer eHealth focus on opportunities to find support from others through online tools, such as online support groups [1-3]. Online support groups may be a particularly attractive means of communication about sensitive prostate cancer topics [3]. They offer the opportunity to maintain anonymity, lurk, cast aside social constraints associated with face-to-face interactions, and interact regardless of location, which some men may find beneficial [3,22].

Although eHealth resources for prostate cancer are abundantly available online, issues concerning the applicability of these eHealth resources across diverse audiences needs further investigation. Given the scarcity of prior work on the specific age-related differences in reasons for using prostate cancer eHealth, we propose the first research question (RQ1): Are there differences between younger and older men living with prostate cancer in reasons for using prostate cancer eHealth for information and support?

### Effects of Using Prostate Cancer eHealth

To date, there are few studies that have focused on the perceived psychosocial effects of using prostate cancer eHealth. Some researchers, such as Dickerson et al [2], report that use of the Internet for prostate cancer information and support enhances the ability to cope with prostate cancer because it helps men feel more informed, in control, and connected with others. Other researchers have begun to evaluate the psychosocial effects of using specific Web-based support tools. For example, Ruland et al [20] found that participants who used the multi-featured illness management tool, WebChoice, had significantly less symptom distress than control group participants. These authors also found within-group improvements in depression within the experimental group.

Not all aspects of prostate cancer eHealth are perceived as having positive effects on psychosocial health. Broom [3] discovered that some men with prostate cancer perceive the anonymity and secrecy of online social support groups, for example, as problematic because unknown, “faceless” individuals may try to deceive them. Men with this perspective generally considered use of this type of eHealth as maladaptive. Expressions of distrust in prostate cancer information found on the Internet has also been found in other studies [2]. Such distrust may be antithetical to the coping process.

Given the lack of a body of research about a range of psychosocial effects of prostate cancer eHealth, consideration of the rigorous literature review of Bjørnes et al [1] about accessibility of prostate cancer information from health care providers and the Internet may be meaningful. Their literature review highlighted words and phrases from the literature that represent “the positive process” of receiving individualized information through dialogue-based contacts with health care providers (ie, the “gold standard”), including words and phrases that reflect positive experiences and feelings, words and phrases that connect these experiences and feelings to the coping process, and how these words and phrases are related to theory (eg, certainty-, security-, and/or empowerment-based theories). These authors also developed a schema of “the negative process” that occurs when information and support are lacking. Words and phrases in the positive dimension included, for example, “being prepared for,” “a sense of confidence and control,” and “coping.” In the negative dimension of the schema, words and phrases included, for example, “suffered in silence and anxiety,” “all alone,” and “fears of the unknown” [1].

Since there is not currently a large body of research to support the effect of prostate cancer eHealth on psychosocial outcomes, investigating men’s perceptions may enhance understanding of the relationship between eHealth and coping. Since perceptions of prostate cancer eHealth might vary by age, the second research question (RQ2) is posed: Are there differences between younger and older men living with prostate cancer in how prostate cancer eHealth affects positive and negative psychosocial outcomes?

## Methods

### Instrumentation

To evaluate use and perceptions of prostate cancer eHealth, survey methodology was used. An online questionnaire was designed using multiple types of response scales for closed-ended questions. For bounded continuous scales, Likert-type scale response anchors as described by Vagias [23] were used with some modifications. The survey was divided into three primary domains of interest in order to address the hypotheses and research questions: (1) Internet behavior and experiences, (2) reasons for using prostate cancer eHealth for information and support needs, and (3) effect on psychosocial indicators. Furthermore, information about personal history and prostate cancer history were assessed to determine the background of the study participants.

To address the third domain, the work of Bjørnes et al [1] was used to develop a measure of how prostate cancer eHealth influences a broad set of psychosocial outcomes. These positive and negative schema were used to inform the development of our measure since the ways in which eHealth influences psychosocial health have not been widely studied. Using their schema, we conceptualized the positive psychosocial dimension to include the following indicators: feeling informed, in control, able to cope, confident about treatment decision, and connected with others. For the negative psychosocial dimension, we conceptualized the indicators as feeling anxious, depressed, lonely, and scared. By using their schema, we hoped to determine whether we could produce a brief yet reliable measure of psychosocial health [1].

### Sampling Strategy and Procedure

Institutional Review Board (IRB) approval for this study was obtained from George Mason University and Inova Health System. Survey participants were recruited using nonprobability sampling methods, including voluntary and snowball sampling. After obtaining permission from website administrators, recruitment occurred through four online prostate cancer social networks—the “New” Prostate Cancer InfoLink Social Network, His Prostate Cancer, the Association of Cancer Online Forums Prostate Problems Mailing List, and a prostate cancer-related email list of Life with Cancer, Inova Health System. All respondents provided informed consent through the questionnaire before the study questions were displayed.

### Measures

#### Sociodemographic and Prostate Cancer Characteristics

Sociodemographic characteristics included questions about age, race/ethnicity, and education level. Race/ethnicity included the answer options “African American/Black,” “Asian/Pacific Islander,” “Hispanic,” “Native American/Alaska Native,” “White,” and “Other”; multiple responses were allowed. Education level was measured using the answer options “high school or less,” “some college,” “college graduate (Bachelor’s degree),” and “graduate degree (Master’s degree or above).” Prostate cancer characteristics were assessed by asking about the amount of time since diagnosis and types of treatment. Time since diagnosis was assessed through the answer options “less than 1 year ago,” “1-2 years ago,” “3-4 years ago,” and “5 years ago or more.” For type of treatment, participants were asked to select all treatments they had received. They could select “prostatectomy,” “radiation (external beam),” “radiation (brachytherapy),” “proton beam therapy,” “hormone therapy,” “testicle removal,” “cryotherapy,” “chemotherapy,” and “watchful waiting.” Other types of treatment not provided as options could be typed in an “other (please specify)” comment field.

#### Internet Behavior and Experiences

Internet measures included questions about men’s Internet behavior and experiences. *Internet use* was measured with the question “how often do you use the Internet?” (1 = never, 2 = almost never, 3 = occasionally, 4 = a moderate amount, 5 = a great deal). *Internet access* was assessed through the following item selections: “I have easy access to the Internet” (1 = strongly disagree, 2 = disagree, 3 = neither agree nor disagree, 4 = agree, 5 = strongly agree). *Level of comfort with the Internet* was measured by “what is your level of comfort when you use the Internet?” (1 = not at all comfortable, 2 = slightly comfortable, 3 = somewhat comfortable, 4 = very comfortable). *Internet use for prostate cancer information* was assessed with “[...] how often have you used the Internet to find information about prostate cancer?” (1 = never, 2 = about once every few months, 3 = about once a month, 4 = about once a week, 5 = about once a day). *Level of difficulty finding information online* was measured by “[...] was it difficult to find the specific information you were looking for?” (1 = never, 2 = almost never, 3 = occasionally, 4 = usually, 5 = always). *Level of applicability of the information* was questioned by “[...] did you think that the prostate cancer information on the Internet applied to your personal situation?” (1 = never, 2 = almost never, 3 = occasionally, 4 = usually, 5 = always). *Trust in online information* was measured by “[...] how much do you trust information about prostate cancer that you get from the Internet?” (1 = never trust, 2 = almost never trust, 3 = occasionally trust, 4 = usually trust, 5 = trust a great deal).

#### Reasons to Use eHealth for Information and Support

Participants were asked why they used eHealth for information and support. They were provided with 13 information categories to select from, such as “to learn more about staging and/or grading,” and five support categories, such as “to read/listen to other men’s personal prostate cancer stories.” Participants could select as many options as applied.

#### Psychosocial Indicators

How the Internet influences psychosocial health was measured with 10 items, such as “I feel informed,” “I feel in control,” and “I feel lonely.” All items were provided with the answer options “more,” “less,” and “no effect.” Scores were assigned to each item by giving a +1 when the Internet had affected men with prostate cancer more, a 0 when the Internet had no effect, and a -1 when the Internet had affected them less. Principle Component Analysis (PCA) with varimax rotation distinguished two reliable components: one for the “positive” effects of eHealth (Eigenvalue [EV] = 1.62, explained variance = 24.23%, alpha = .70) and one for the “negative” effects of eHealth (EV = 3.59, explained variance = 27.92%, alpha = .83). Two sum scales were computed, one representing the positive effects of eHealth and one representing the negative effects of eHealth.

### Statistical Analysis

We used descriptives and chi-square statistics to present the sociodemographic and prostate cancer characteristics. To address the first research domain, we tested whether there were differences between men under 65 years old and men 65 years old and older in Internet behavior (H1a) and experiences (H1b). Analysis of variance (ANOVA) tests were conducted with age group as the independent variable and the seven Internet measures as dependent variables. For the purpose of investigating the second domain, we used chi-square statistics to examine the differences between men under 65 years old and men 65 years old and older in reasons for using eHealth information to address information and support needs (RQ1). To investigate the third and final domain, differences between men under 65 years old and men 65 years old and older in how prostate cancer eHealth impacts psychosocial indicators (RQ2) were examined using Kendall’s tau-b correlation coefficients. The relationships between age, Internet measures, and psychosocial indicators were further explored using the conditional process modeling program PROCESS, Model 4 [24]. All indirect effects were subjected to bootstrap analyses with 5000 bootstrap samples and a 95% CI.

## Results

### Sociodemographic and Prostate Cancer Characteristics

A total of 402 respondents started the online survey, of which 382 completed the survey (completion rate = 95.0%). Another 93 participants out of 382 (24.3%) chose not to fill out their age, and therefore were excluded from the data as we were not able to analyze age differences in use and perceptions of eHealth information for this group. This resulted in 289 valid cases for data analysis. Our sample of men with prostate cancer were on average 64.91 years old (SD 8.34, range 40-89). Most participants were white (277/289, 95.8%), and almost half of them had a graduate degree (134/289, 46.4%). For analysis, the sample was divided into a cohort of younger men (40-64 years old, 144/289, 49.8%) and older men (≥ 65 years old, 145/289, 50.2%). Older men were more likely to be diagnosed five years ago or more (χ^2^_1_= 13.3, *P*<.001), whereas younger men were more likely to be diagnosed less than one year ago (χ^2^_1_= 8.5, *P*=.004). In terms of treatments men had undergone, younger men were more likely to have had a prostatectomy than older men (χ^2^_1_= 13.9, *P*<.001) and older men were more likely to have had hormone therapy than younger men (χ^2^_1_= 3.8, *P*=.05). Table 1 shows an overview of results related to personal and prostate cancer characteristics.

**Table 1:** Personal and prostate cancer characteristics (n=289)^a^.

Characteristics		Younger men (< 65 years), n (%), mean (SD), or range	Older men (≥ 65 years), n (%), mean (SD), or range
Number of men per age group, n (%)		144 (49.8)	145 (50.2)
Age in years, mean (SD)		58.28 (4.62)	71.49 (5.51)^b^
Age in years, range		40-64	65-89
**Ethnicity, n (%)**			
	White	135 (93.8)	142 (97.9)
	African American/Black	4 (2.8)	2 (1.4)
	Asian/Pacific Islander	2 (1.4)	3 (2.1)
	Hispanic	2 (1.4)	2 (1.4)
	Native American/Alaska Native	2 (1.4)	0 (0)
**Education level, n (%)**			
	High school or less	8 (5.6)	7 (4.8)
	Some college	28 (19.4)	35 (24.1)
	College graduate (Bachelor’s degree)	43 (29.9)	33 (22.8)
	Graduate degree (Master’s degree or above)	65 (45.1)	69 (47.6)
**Time since diagnosis, n (%)**			
	Less than 1 year ago	36 (25.0)	16 (11.0)^c^
	1-2 years ago	40 (27.8)	32 (22.1)
	3-4 years ago	36 (25.0)	34 (23.4)
	5 years ago or more	32 (22.2)	62 (42.8)^b^
**Type of treatment, n (%)**			
	Prostatectomy	79 (54.9)	47 (32.4)^b^
	Hormone therapy	41 (28.5)	58 (40.0)^d^
	Radiation—external beam	39 (27.1)	54 (37.2)
	Watchful waiting/active surveillance	25 (17.4)	36 (24.8)
	Chemotherapy	11 (7.6)	10 (6.9)
	Radiation—brachytherapy (implants)	10 (6.9)	19 (13.1)
	Proton beam therapy	3 (2.1)	7 (4.8)
	Cryotherapy	1 (0.7)	4 (2.8)
	Testicle removal	0 (0)	0 (0)

^a^Some numbers do not add up to 100% due to missing data.

^b^Differs significantly from younger men (*P*<.001).

^c^Differs significantly from younger men (*P*=.004).

^d^Differs significantly from younger men (*P*=.05).

### Domain 1: Internet Behavior and Experiences

To describe our findings regarding the first research domain, we found that the two age groups significantly differed on the frequency of Internet use (H1a) (*F*_1,285_= 3.80, *P*=.05, η_p_^2^ =.01) and Internet experiences (H1b), such as level of comfort with the Internet (*F*_1,286_= 6.31, *P*=.01, η_p_^2^ = .02). The means show that men in the older cohort used the Internet less frequently than men in the younger cohort and also felt less comfortable using the Internet, confirming our hypothesis (see Table 2).

**Table 2:** Internet behavior and experiences among younger (< 65 years) and older (≥ 65 years) men with prostate cancer.

Internet behavior and experiences^a^	Younger men (< 65 years), mean (SD)	Older men (≥ 65 years), mean (SD)
Internet use	4.84 (0.39)	4.74 (0.47)^b^
Internet access	4.60 (1.00)	4.54 (0.88)
Internet comfort^c^	3.85 (0.39)	3.72 (0.49)^d^
Internet use for prostate cancer information	3.78 (1.10)	3.81 (1.09)
Information-seeking difficulty	2.57 (0.96)	2.58 (0.88)
Internet personal applicability	3.67 (0.67)	3.59 (0.66)
Internet trust	3.76 (0.66)	3.68 (0.66)

^a^All measures were assessed using a 5-point Likert-type scale.

^b^Differs significantly compared to younger men (*P*=.05).

^c^Level of comfort with the Internet was measured on a 4-point Likert scale.

^d^Differs significantly compared to younger men (*P*=.01).

### Domain 2: Reasons to Use eHealth for Information and Support Needs

The second domain investigated (RQ1) showed that the most frequently selected reasons to address information needs were to learn more about available treatments (255/289, 88.2%), to learn more about the effects of treatment (245/289, 84.8%), and to keep up to date with prostate cancer research (237/289, 82.0%). We found that men in the younger cohort used the Internet more often to be informed about treatment options (χ^2^_1_= 4.4, *P*=.04) and to learn more about staging/grading (χ^2^_1_= 7.7, *P*=.01) than men in the older cohort. Our results showed that the most common reasons to use eHealth to address support needs were to read and/or listen to other men’s prostate cancer stories (192/289, 66.4%), to offer their own personal prostate cancer stories (136/289, 47.1%), and to get personal opinions to help make a treatment decision (135/289, 46.7%). Our results revealed that men in the younger cohort used the Internet significantly more often than older men to get emotional support and encouragement online (χ^2^_1_= 12.0, *P*=.001). Table 3 provides an overview of the information and support needs.

**Table 3:** Reasons to use eHealth for information and support needs among younger (< 65 years) and older (≥ 65 years) men with prostate cancer.

Reasons to use eHealth^a^	Younger men (<65 years) (n=144), n (%)	Older men (≥65 years) (n=145), n (%)
To learn more about available treatments	129 (89.6)	126 (86.9)
To learn more about the effects of treatment	122 (84.7)	123 (84.8)
To keep up to date with prostate cancer research	115 (79.9)	122 (84.1)
To learn more about recurrence of prostate cancer	101 (70.1)	98 (67.6)
To be informed about treatment options	114 (79.2)	98 (67.6)^b^
To know what questions to ask my doctor	112 (77.8)	101 (69.7)
To learn more about staging and/or grading	113 (78.5)	93 (64.1)^c^
To learn more about self-management	79 (54.9)	87 (60.0)
To make sure what the doctor told me is correct	66 (45.8)	66 (45.5)
To make a treatment decision using a website tool	54 (37.5)	53 (36.6)
To check out my doctor’s reputation	52 (36.1)	44 (30.3)
To get a second opinion	39 (27.1)	55 (37.9)
To learn more about and/or enroll in a clinical trial	41 (28.5)	41 (28.3)
To read/listen to other men’s prostate cancer stories	96 (66.7)	96 (66.2)
To offer my personal prostate cancer story	64 (44.4)	72 (49.7)
To get personal opinions to help decision making	64 (44.4)	71 (49.0)
To get personal opinions to help address treatment effects	58 (40.3)	69 (47.6)
To get emotional support and encouragement	48 (33.3)	22 (15.2)^d^

^a^More than one reason to use eHealth for information needs could be selected. Reasons are presented from most frequently selected reasons to least frequently selected reasons.

^b^Percentage differs significantly compared to younger men (*P*=.04).

^c^Percentage differs significantly compared to younger men (*P*=.01).

^d^Percentage differs significantly compared to younger men (*P*=.001).

### Domain 3: Impact on Psychosocial Indicators

Examining the third and final domain (RQ2), we found that increasing age was positively related to negative psychosocial indictors, indicating that older men with prostate cancer were more likely to feel lonely, depressed, anxious, and scared as a result of using the Internet for prostate cancer eHealth than men in the younger cohort (tau-b=.17, *P*=.002). We found that positive psychosocial indicators were positively related to Internet use (tau-b=.16, *P*=.004), Internet use for prostate cancer (tau-b=.14, *P*=.005), personal applicability of the Internet (tau-b=.15, *P*=.004), and Internet trust (tau-b=.21, *P*<.001). This indicates that more frequent use of the Internet, personally relevant information on the Internet, and higher trust in the Internet might result in a more positive experience of using the Internet. Furthermore, positive psychosocial indicators were negatively related to the level of difficulty in use of the Internet (tau-b=-.12, *P*=.02), indicating that the easier it is to use the Internet, the more positive experience men have with the Internet. In addition, negative psychosocial indicators were significantly and negatively related to Internet trust (tau-b=-.11, *P*=.04), suggesting that less trust in Internet information may lead to a more negative experience of the Internet. Factor loadings for psychosocial indicators are displayed in Table 4 and correlation coefficients in Table 5.

**Table 4:** Factor loadings for psychosocial indicators.

Psychosocial indicators^a^	Component 1, *r*	Component 2, *r*
I feel in control	*.68* ^b^	-.19
I feel like I can cope	*.63*	-.33
I feel connected with others living with prostate cancer	*.63*	-.02
I feel connected with my spouse/partner	*.60*	.01
I feel confident about my treatment decision	*.60*	-.23
I feel informed	*.59*	-.09
I feel scared	-.08	*.85*
I feel depressed	-.13	*.84*
I feel lonely	-.10	*.83*
I feel anxious/stressed	-.26	*.69*

^a^Negatively phrased items were not reversely recoded as Principle Component Analysis (PCA) distinguished the same two scales and same factor loadings when using the negatively phrased items.

^b^Italic numbers indicate which items load onto which components.

**Table 5:** Correlations between age, Internet measures, and psychosocial indicators.

Age, Internet measures, and psychosocial indicators	Correlations between age, Internet measures, and psychosocial indicators, Kendall's tau-b^a^									
	1.	2.	3.	4.	5.	6.	7.	8.	9.	10.
1. Age^b^	-									
2. Positive dimensions	-.08	-								
3. Negative dimensions	.17^c^	-.31^d^	-							
4. Internet use	-.12^e^	.16^f^	.04	-						
5. Internet access	-.12^g^	.07	0	.33^d^	-					
6. Internet comfort	-.16^h^	.10	-.01	.44^d^	.37^i^	-				
7. Internet use for prostate cancer information	.01	.14^h^	-.05	.14^h^	.09	.07	-			
8. Information-seeking difficulty^j^	0	-.12^k^	-.02	-.05	-.08	-.13^k^	0	-		
9. Internet personal applicability	-.04	.15^f^	-.07	.06	.15^h^	.06	.04	-.32^d^	-	
10. Internet trust	-.05	.21^d^	-.11^g^	.03	.13^l^	.09	0	-.18^i^	.42^d^	-

^a^Correlation coefficients are Kendall’s tau-b coefficients for ordinal level variables.

^b^Age as dichotomous variable. Using the continuous variable of age resulted in the same results.

^c^The correlation was significant (*P*=.002).

^d^The correlation was significant (*P*<.001).

^e^The correlation was significant (*P*=.05).

^f^The correlation was significant (*P*=.004).

^g^The correlation was significant (*P*=.04).

^h^The correlation was significant (*P*=.01).

^i^The correlation was significant (*P*=.001).

^j^The higher the score, the more difficult information seeking was perceived.

^k^The correlation was significant (*P*=.02).

^l^The correlation was significant (*P*=.03).

**Figure 1:**
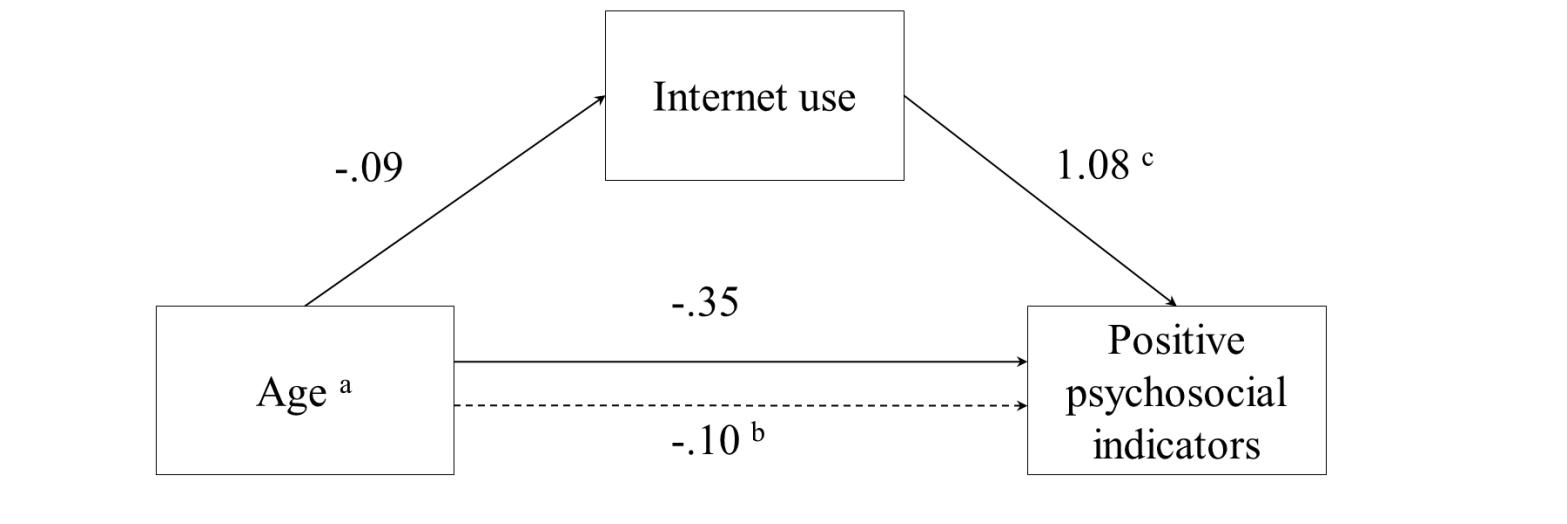
Mediation model: The effect of age on positive psychosocial indicators through Internet use. Unstandardized regression coefficients are presented. (a) Age as dichotomous variable. Using the continuous variable of age resulted in the mediation effect. (b) Significant at 95% CI -0.28 to 0. (c) P=.001.

When further exploring the relationships between age, Internet measures, and psychosocial indicators, we found a significant negative mediated effect of age on the positive psychosocial dimension through Internet use. The model showed an insignificant direct effect of age on positive psychosocial indicators (*b*=-0.35, *P*=.17), but a significant indirect effect of age on the positive psychosocial dimension via Internet use (*b*=-0.10, 95% CI -0.28 to 0). This suggests that older men use the Internet less than their younger counterparts, which causes them to have a less positive experience when using the Internet (see Figure 1).

## Discussion

### Principal Findings

Our findings show that there may be age-related differences in use and perceptions about prostate cancer eHealth information and support among men with prostate cancer. Perhaps most provocative, when men were asked how prostate cancer information and/or support found on the Internet affected them, men in the older cohort were more likely to report that it made them feel lonely, depressed, anxious/stressed, and scared, for example (negative psychosocial indicators). We also found significant positive associations between measures of Internet use and, for instance, feeling informed, in control, and confident about treatment decision (positive psychosocial indicators). Moreover, we found that Internet use mediated the association between age and the positive dimension of psychosocial indicators, which shows that greater use of the Internet among men in the younger cohort, in particular, appears to lead to a more positive psychosocial response to prostate cancer eHealth. That men in our younger cohort were significantly more likely to use the Internet and feel comfortable with using the Internet is consistent with findings from past investigations [13,25].

We also explored whether our two cohorts would differ in reasons for using prostate cancer eHealth. We found that, when compared to older men, men in the younger cohort used eHealth information significantly more to be informed about treatment options and to learn more about staging and/or grading. Additionally, younger men reported using communication for emotional support and encouragement significantly more often than older men. Although a significant difference in time since diagnosis between the cohorts might partially explain this finding—older men had a longer time since diagnosis—it is still worthy of attention. Dickerson et al [2] describe online social support as an “online friendship.” It is possible that such friendships can provide emotional support and encouragement, and in a format that younger men are comfortable with navigating as experienced Internet users. Because men under 65 years old make up a relatively smaller proportion of the prostate cancer population, it is possible that they have a more difficult time finding support in their own social circles when compared to older aged men. Online social networks may offer a way to generate new social circles that would not have been possible prior to the existence of nonstatic Web technologies [26]. Our study may have uncovered an important distinction in the eHealth needs of men with prostate cancer who are diagnosed at younger ages, a rapidly growing segment of the prostate cancer population [8], however, further investigation is needed.

### Study Limitations

Although our findings shed light on the fact that there may be age-related differences in the use of prostate cancer eHealth and perceptions about how it affects one’s psychosocial health, these results should be interpreted with caution. Since there was a significant difference between cohorts in time since diagnosis (longer time since diagnosis for the older cohort) and types of treatment regimens (greater frequency of hormone therapy over prostatectomy for the older cohort), our findings might have detected differences in use and perceptions based on time since diagnosis or treatment regimen. For example, men diagnosed longer ago may use certain features of eHealth less or more frequently, which was not measured in this study. Furthermore, treatment effects on psychosocial health, such as depression or anxiety, as well as baseline predispositions related to depression, anxiety, and coping ability may have influenced participants’ responses about the specific effect of eHealth on their psychosocial health. Finally, certain treatment effects, such as cognitive effects associated with hormone therapy, could have influenced findings related to use and perceptions of prostate cancer eHealth [27].

Other limitations of this study included those related to selection bias. While the sample was fairly representative of the prostate cancer population based on age distribution [6], the findings are not generalizable to the entire prostate cancer population since the survey sample was predominantly non-Hispanic white, well-educated men, with easy access to the Internet. Because our sampling strategy involved voluntary recruitment of men from prostate cancer social networks, it is not surprising that both cohorts were relatively frequent and comfortable Internet users. It may also explain why we did not detect significant differences between cohorts for several measures of Internet behavior or experiences, such as ease of access to the Internet, frequency of use of the Internet to seek prostate cancer eHealth, and level of trust in prostate cancer eHealth. Furthermore, as we dealt with cross-sectional data, we can only suggest that Internet behavior and experience may lead to positive or negative psychosocial experiences as a result of using eHealth. It could also be the case that, for instance, due to negative psychosocial experiences with prostate cancer eHealth, men trust the Internet less, and therefore use the Internet less as a source of information and support.

### Implications and Directions for Future Research and Practice

There are several implications of this study for future research and practice. As described by Harden et al [28] and reiterated by Bjørnes et al [1], men with prostate cancer have a great deal of information and support needs, but each man needs different information or needs the information to be presented differently. Our study findings show that eHealth information and support needs for prostate cancer may vary by age, in particular. With respect to tailoring of future eHealth interventions, men under 65 years old may benefit from nonstatic Web technologies so that they can receive ample emotional support and encouragement in addition to informational support. In turn, men 65 years and older may benefit from assistance with using the Internet in more advanced ways, since increased Internet experience and comfort with use may promote positive psychosocial effects, such as feeling more in control and informed about prostate cancer. Nevertheless, future research is needed to unravel age-related factors underlying age-related differences to be better able to tailor prostate cancer eHealth information to men’s information and support needs.

Kreps [29] describes the importance of audience analysis to better meet audience needs related to Internet information technologies. With this recommendation in mind, future prostate cancer eHealth studies that build on these study findings should not only analyze men by age, but also by ethnicity. Whether or not men who were underrepresented in this study use and perceive prostate cancer eHealth in the same way as their non-Hispanic, white counterparts remains in question. We particularly recommend that future studies include a representative sample of men from different racial backgrounds, particularly African-American/black men given their two-fold increased risk for prostate cancer when compared to white men. We also suggest inclusion of other population segments that may be impacted by the “digital divide,” such as men with different levels of education and income, and those who live in urban versus rural areas [30]. Most importantly, the understanding and appreciation of diverse audience segments gleaned from research should be used to inform translation of evidence to practice.

## Abbreviations

ANOVA: analysis of variance
EV: Eigenvalue
H1a: Hypothesis 1a
H1b: Hypothesis 1b
IRB: Institutional Review Board
PCA: Principle Component Analysis
RQ1: research question 1
RQ2: research question 2
